# Managing public–private partnerships for health diagnostics: challenges and strategies

**DOI:** 10.1093/heapol/czag041

**Published:** 2026-03-27

**Authors:** Sara Bennett, Jennifer Applegate, Brenda Bunyasi, Adanna Uloaku Nwameme, Noelle Orata, Abigail Neel, Ligia Paina, Adjeiwa Akosua Affram, Daniel Nana Yaw Abankwah, Mohammed Abseno, Daniela C Rodriguez, Getnet Mitike, Justice Nonvignon, Francis Wafula

**Affiliations:** Department of International Health, Johns Hopkins Bloomberg School of Public Health, 615 N Wolfe St, Baltimore, MD 21205, United States; Department of International Health, Johns Hopkins Bloomberg School of Public Health, 615 N Wolfe St, Baltimore, MD 21205, United States; Prism Centre, Institute of Health Care Management, Strathmore University, Ole Sangale Rd, Madaraka, PO Box 59857-00200, Nairobi, Kenya; Department of Social and Behavioral Sciences, School of Public Health, University of Ghana, LG13, Legon 381, Ghana; Prism Centre, Institute of Health Care Management, Strathmore University, Ole Sangale Rd, Madaraka, PO Box 59857-00200, Nairobi, Kenya; Department of International Health, Johns Hopkins Bloomberg School of Public Health, 615 N Wolfe St, Baltimore, MD 21205, United States; Department of International Health, Johns Hopkins Bloomberg School of Public Health, 615 N Wolfe St, Baltimore, MD 21205, United States; Department of Health Policy, Planning and Management, School of Public Health, University of Ghana, LG13, Legon 381, Ghana; Department of Health Policy, Planning and Management, School of Public Health, University of Ghana, LG13, Legon 381, Ghana; International Institute for Primary Health Care, Gulelle Arbegnoch St, Addis Ababa 1242, Ethiopia; Department of International Health, Johns Hopkins Bloomberg School of Public Health, 615 N Wolfe St, Baltimore, MD 21205, United States; International Institute for Primary Health Care, Gulelle Arbegnoch St, Addis Ababa 1242, Ethiopia; Department of Health Policy, Planning and Management, School of Public Health, University of Ghana, LG13, Legon 381, Ghana; Department of International Health, Johns Hopkins Bloomberg School of Public Health, 615 N Wolfe St, Baltimore, MD 21205, United States

**Keywords:** diagnostics, public–private partnership, Africa, management, process evaluation

## Abstract

This paper draws upon a process evaluation of a public–private partnership (PPP) for diagnostics in three Sub-Saharan African countries, Ethiopia, Ghana and Kenya. The study sought to identify challenges in managing health PPP projects and potential solutions. We used an extensive document review and 72 recent key informant interviews (KIIs), building on 63 KIIs previously conducted to analyze the African Health Diagnostics Platform (AHDP) project in-depth. Our analysis employed a framework developed by Magalhaes et al based on the broader (non-health) PPP literature that identifies key challenges, strategies and success factors in PPP management across three main stages of PPP implementation. We find considerable alignment between the management challenges identified in the broader PPP literature and AHDP. Certainly, intensive negotiations and high transaction costs; difficulties managing risks and financing; the need for highly complex planning; and challenging stakeholder management all played a role in slowing progress on AHDP. An additional, critical theme concerns lack of capacity for managing health PPPs. The AHDP project generated a number of innovations to facilitate management but overall, if health PPPs are to succeed, more needs to be done to support their implementation. In particular, we propose investment in training government and technical assistance providers in health PPPs; development of repositories of guidance documents to support health PPPs; employment of systems-thinking based planning approaches that illuminate connections across the health system; more sophisticated approaches to stakeholder management; and investment in research that supports modeling of different PPP arrangements and how their progression is influenced by local contextual factors. While evidence on the impact of health PPPs remains scarce, moves toward increased healthcare corporatization in the context of dwindling aid underscores the urgency of building experience and evidence on PPPs in healthcare and other social sectors.

Key messages

- Public–private partnerships (PPPs) for health often struggle to take off due to the complex managerial demands that they impose on project partners.
- Successful healthcare PPPs require intense negotiations, careful management of risk and financial arrangements, complex planning, and sophisticated stakeholder management.
- Currently, capacity to manage health PPPs is lacking in government and the broader global health community. In light of moves towards increasing corporatization of health investments, capacity and evidence are both needed urgently.

## Introduction

There is substantial debate on the potential role and impact of public–private partnerships (PPPs) in healthcare. PPPs have potential to improve service delivery through improved availability of infrastructure, equipment and supplies, strengthening operational processes and developing the skills of health staff (Cohen 2016, Kebede et al. 2016, Joudyian et al. 2021, Ravishankar and Lehmann n.d.). These arguments have been applied to varying aspects of healthcare, from discrete services like hemodialysis (Paltiel et al. 2020) and radiotherapy (Anakwenze Akinfenwa et al. 2021), to primary health care and whole hospital systems (Vian et al. 2015, Scott et al. 2022).

Clinical diagnostic services have been flagged as one area where PPPs could deliver value (Kebede et al. 2016, Shrivastava et al. 2016). Public sector diagnostic services in Africa are typically provided within healthcare facilities. Recent reviews suggest under-resourcing and suboptimal performance, with low availability of essential diagnostic tests as a consequence (Schroeder and Amukele 2014, Fleming et al. 2021, Yadav et al. 2021), resulting in symptomatic diagnosis, or patients seeking diagnostic services at more costly private facilities with indeterminable quality (Elbireer et al. 2013), thus contributing to suboptimal treatment processes and outcomes, and increased danger of antimicrobial resistance.

A PPP is broadly defined as a long-term contractual arrangement between a public contracting authority and a private partner, where the latter undertakes a public function on behalf of the government who in turn compensates the private partner based on predefined milestones (Torchia et al. 2015). While definitions vary, PPPs share certain features, including lengthy contract periods (10–30 years), transfer of some risk from the public authority to the private partner, and typically, transfer of assets or expertise from the private entity to government at the end of the contract period. While there are many collaborative arrangements between public and private entities, most do not meet the strict definition of a PPP (Roehrich et al. 2014) having limited or no private financing, and minimal risk transfer to the private partner.

Despite calls to introduce healthcare PPPs across low- and middle-income countries (LMICs), evidence on their role, performance and impact remains scant, especially in Sub-Saharan Africa (Roehrich et al. 2014). Most of what we know of health PPPs in Sub-Saharan Africa draws from the Queen Mamohato Memorial Hospital PPP in Lesotho (Vian et al. 2015, Scott et al. 2022). Despite improving service availability and access, the PPP prematurely terminated, reportedly due to late payments by government, that contributed to stakeholder conflict (‘Netcare responds to Tsepong Issues’ 2021). Evidence from multiple contexts points to management and implementation challenges of this nature undermining PPPs, with management challenges stemming from limited public-sector capacities especially for health PPPs and complex policy frameworks (Ovcharova and Grabowska 2022, Dove et al. 2025).

While there is limited experience with health PPPs, there is substantially more in other sectors (e.g. infrastructure, energy). What is unclear is the extent to which lessons from such capital-intensive projects can be transferred to service-heavy sectors like healthcare. Analysts have synthesized lessons on management of a wide array of PPPs. Magalhães et al., for example, drew upon a systematic review to identify challenges, strategies and success factors in PPP management across three implementation stages: Pre-project (from idea to contract), On-project (from contract signature to close out), and Post-project (after close out of contract) (Magalhães et al. 2020). Table 1 identifies the key domains presented by Magalhães et al.

**Table 1:** Summary of PPP project management challenges, strategies and success factors as described by Magalhães et al (2020).

	Construct & definition	Challenges	Strategies	Success factors
Pre-Project	**Transaction costs**—time & effort spent on finding contractors, researching options, getting quotes, negotiating terms and finalizing the contract	High transaction costs (extensive negotiations)	Well-structured process for evaluating bidders Well defined scope in contract	
	**Risk, Vulnerabilities & Financing**—potential events that could affect project viability, underlying weaknesses that increase exposure to these risks and funding for the project	Allocation of risk between main stakeholders Flawed risk assessment Neglect of project vulnerabilities	Modeling of risk between partners Appropriate risk allocation Vulnerability analysis	Appropriate risk and financing estimates for the project
	**Market conditions**—the economic, financial & industry environment and how it affects the private sector’s appetite for PPPs	Lack of favorable economic environment	Establish support mechanisms to foster national market around PPPs	Economic viability and decent rate of return on project Favorable investment environment
On-Project	**Risk, Vulnerabilities & Financing**—potential events that could affect project viability, underlying weaknesses that increase exposure to these risks and funding for the project	Flaws in project models and estimations Unexpected political and economic vulnerabilities Ensuring adequate funding to sustain project	External reviews of planning and project models Regulatory frameworks to minimize risks from political and economic instability	
	**Planning**—coordination of the technical, financial and institutional aspects of the project to ensure it is delivered and operated according to the PPP terms	Poor project planning that does not match project complexity	Rigorous planning e.g. work breakdown structure Maintain project management team with high level leadership Clear metrics, goals and outcomes to guide teams	Proper project preparation with clear goals and outcomes Appropriate quality management
	**Stakeholder Management**—ongoing process of identifying, engaging and coordinating stakeholders to manage expectations, address concerns and support effective PPP delivery	Weak stakeholder engagement and management (leads to resistance to project) Poor relationship between public and private actors and poor relationship management	Deployment of tools to manage stakeholder relationships	Continuous involvement of stakeholders Level of trust between parties
	**Organizational culture**—shared values, norms, behaviors and ways of working, and the degree of alignment between public and private sectors	Clash of internal cultures (public/private) and ways of working		Level of trust between parties Alignment of company culture with expectations

PPPs, public–private partnerships.

This paper uses a process evaluation of a diagnostics PPP in three Sub-Saharan African countries (Ethiopia, Ghana, and Kenya) to identify challenges in structuring and managing PPP projects for health, and possible remedial strategies. We also seek to understand similarities and differences between project management of health sector PPPs versus other types of PPPs. We provide an overview of the African Health Diagnostics Platform (AHDP) project, describe our methods, and present findings (challenges and potential remedial strategies) using the themes identified by Magalhães et al.

### Introduction to the project

AHDP was conceived and funded by the Gates Foundation to address the problem of inadequate access to quality diagnostics in Africa through laboratory diagnostics PPPs. The project combined technical assistance to governments by the Clinton Health Access Initiative (CHAI) and packages of loan-based international financing provided by the European Investment Bank (EIB). The initial project theory of change indicated that technical assistance would enable governments to design, implement and monitor diagnostic PPPs, supported by AHDP’s international financing mechanisms like concessionary loans to governments and private firms to cover large capital outlays. International financing also aimed to address financial risks faced by private firms stemming from, for instance, unreliable government payments through the provision of sovereign guarantees. Diagnostic PPPs were envisaged to improve clinical laboratory management, enhance transport and referral networks for diagnostics, enlarge the test menu, ensure availability of diagnostics, and reduce test turnaround times.

Funding partners set an ambitious timeline for the project, anticipating that contracts would be in place and services delivered within 3 years. Actual progress on implementation was significantly slower (Table 2). At the point of final data collection (mid-2024), a tender had not yet been issued in Ghana, whereas the Ethiopian Government had just finished reviewing proposals submitted under a Request for Proposals (RFP) call and was moving toward announcing the winning bidder. In Kenya, a PPP project in Tharaka Nithi (one of Kenya’s 47 counties) had begun implementation, but progress toward establishing PPPs in other counties had stagnated. Citing frustration with the slow uptake of PPPs in the three countries, the Gates Foundation ceased AHDP support in December 2023.

**Table 2:** Timeline of the African health diagnostics platform project.

Date	Step/event
**2016**	Consultancy studies inform Gates Foundation understanding of scope to centralize and strengthen lab services in Africa
**2017**	Gates Foundation explores EIB interest in collaboration through the European Fund for Sustainable Development
**January 2018**	Gates Foundation and the European Commission announce formal collaboration
**Jan–June 2018**	McKinsey contracted to define and plan diagnostic PPPs in two countries.
**July 2019**	First 13-month contract awarded to CHAI to launch AHDP
**2019–2021**	CHAI conducts feasibility studies to evaluate technical and financial feasibility and assess market demand
**March 2020**	COVID-19 pandemic declared
**July–Aug 2020**	Additional funding awarded to CHAI to support AHDP including: Support for roll out in Ethiopia, Ghana, Kenya and Rwanda^a^ (July 2020–Dec 2023); Support to ‘Closing the Loop’ investment for Sickle Cell in Ghana and Hepatitis C in Rwanda (2020–22) to demonstrate impact of improved testing on services
**October 2020**	Funding awarded to JHU for evaluation and learning
**2020**	Ghana passes Act 1039, the Public Private Partnership Act which outlines procedures and requirements for entering into PPP agreements
**September 2021**	Tharaka Nithi County, Kenya request for Expression of Interest issued for Clinical Laboratory Improvement Project
**January 2022**	Tharaka Nithi County, Kenya Bidder briefing and Selection of Bidder
**December 2022**	First Request for Quotations issued in Ethiopia—Failed due to only one qualified bidder (PPP law requires min of 3 qualified bidders)
**January 2023**	Tharaka Nithi County, Kenya contract negotiated and signed
**April 2023**	Gates Foundation, EIB, CHAI, and JHU participate in a joint learning trip to Kenya and Ethiopia to meet with key stakeholders for AHDP
**March 2023**	Ghana’s draft RFQ for the AHDP PPP project submitted to Ministry of Finance for approval
**September 2023**	Ghana develops revised scope for contract to implement AHDP only in 8 regional sites
**September 2023**	Ethiopia adjusted PPP model to focus on 6 federal hospitals due to jurisdictional complexities and financial feasibility
**October 2023**	Ethiopia redesigns and reissues RFQ for Integrated Diagnostic Center
**October 2023**	Gates Foundation makes the decision to not extend CHAI’s funding to provide technical assistance for Phase I AHDP countries.
**December 2023**	Gates Foundation support to CHAI ends
**January 2024**	CHAI concludes work for the Foundation on AHDP, though continues to provide some support to country governments without funding support.
**March 2024**	Ethiopia RFP published and shared with three qualified bidders
**April 2024**	Tharaka Nithi, Kenya PPP Operationalization (soft launch)
**July 2024**	Ethiopia evaluation of request for proposals completed, winning bid shared with PPP Board for approval
**December 2024**	Ethiopia—government approval of the winning consortium’s bid provided in writing

AHDP, African Health Diagnostics Platform; CHAI, Clinton Health Access Initiative; EIB, European Investment Bank; JHU, Johns Hopkins University; PPP, public–private partnership.

^a^Rwanda was initially included as one of the target countries for AHDP but decided not to proceed with the project quite early. It is not included in the analysis presented here.

## Methods

### Evaluation design

This was a prospective, mixed-methods study combining a process and outcome evaluation, implemented through a consortium of four research partners. While the outcome evaluation proceeded in just one county in Kenya (the only jurisdiction that successfully launched an AHDP project during the study period), the process evaluation was carried out across all three countries to understand the process, including barriers. This paper draws upon the process evaluation alone.

### Data sources

#### Document review

A document review was initiated at the start of the evaluation, encompassing previous assessments on diagnostics, background information on the AHDP project, and PPP design considerations, including feasibility, anticipated impact, and potential risks. The review started in 2021 and continued throughout the duration of the evaluation as additional documents became available, including tender-related documents and program reports. National policies and guidelines relevant to the PPP were also reviewed. Over 136 documents were identified and reviewed across the three countries (15 general; 44 Ghana; 38 Ethiopia; 39 Kenya).

#### Key informant interviews (KIIs)

Two rounds of KIIs were conducted with selected respondents who were either familiar with AHDP discussions and implementation, or were in decision-making roles related to PPPs. The first round of KIIs (*n* = 63) was conducted between November 2021 and January 2022 and written up as an interim report. The second round (*n* = 72) conducted between July and October 2024, built on previous findings. While this paper primarily reports second round findings, it draws on insights developed during the first round. Table 3 shows the profile of interview respondents.

**Table 3:** Key informant interview (KII) respondent characteristics round 2 interviews.

	Female	Male	Total	Ethiopia	Ghana	Kenya	Global	Total
**Government**								
**Government, national (MOH, MOF)**	4	11	**15**	5	9	1	0	**15**
**Government, subnational (e.g. district & county officials)**	0	4	**4**	4	0	0	0	**4**
**Health facility staff**	7	12	**19**	7	0	12	0	**19**
**Private**								
**Private companies**	3	4	**7**	6	0	0	1	**7**
**Implementing partners**								
**Technical assistance partner (CHAI)**	6	5	**11**	1	1	2	7	**11**
**Other implementing partners**	3	2	**5**	3	2	0	0	**5**
**Financing partners**								
**Gates Foundation & EIB**	2	4	**6**	0	0	0	6	**6**
**Other**								
**Professional associations**	1	4	**5**	2	3	0	0	**5**
**Total**	**26**	**46**	**72**	**28**	**15**	**15**	**14**	**72**

The figures in bold are totals. CHAI, Clinton Health Access Initiative; EIB, European Investment Bank; MOH, Ministry of Health; MOF, Ministry of Finance.

Interviews were primarily conducted in English, in-person or over Zoom. Oral informed consent was obtained prior to the interview. If the respondent was uncomfortable being recorded, then detailed notes were taken. For interviews conducted in English, audio files were transcribed. A small number of interviews were conducted in Amharic and transcribed and translated into English.

The study was approved by the Institutional Review Board of the main grant recipient, (where it was determined to be non-human subjects’ research) and by institutional review boards in all three study countries.

### Analysis

An iterative approach was taken for data collection and analysis. The initial codebook developed was broad and atheoretical including codes on Actors (e.g. issues of power, motivation & relationships); Health systems and government policies (e.g. lab systems, human resources, health financing, and government policies); Dynamics around the PPP (e.g. issues arising during negotiations, anticipated threats); Learning; PPP design and negotiation; PPP procurement and implementation; and Sustainability. This codebook was tested and refined across the evaluation consortium, and subsequently applied to all interview transcripts. Throughout the interviewing and coding processes, debriefing sessions were held within and across study teams to discuss emerging themes and identify cross-cutting issues. We conducted separate thematic analyses for global and country-level interviews but used a common codebook on the Atlas.ti platform. Overarching lessons learned were developed and refined collaboratively by consortium members through ongoing debriefing sessions, and two cross-consortium workshops in October and November 2024. Finally, evaluation findings were validated with government officials in each study country. The PPP project management framework (Magalhães et al. 2020) used to structure this paper was identified and applied post-hoc as a means to organize the main themes from our findings.

## Findings

Our findings describe the pre-project themes for all three countries and on-project considerations for Ethiopia and Kenya, the two countries that proceeded to implementation. Pre-project dimensions explored include transaction costs; risks, vulnerabilities and financing; as well as market conditions. On-project dimensions include risks, vulnerabilities and financing; planning; stakeholder management and organizational culture.

### Transaction costs

#### Challenges

Across all three countries, the level and intensity of pre-project negotiation was considerably greater than originally planned for. High transaction costs arose for a number of reasons including the need to navigate highly structured PPP Acts, the lack of familiarity of actors with these Acts and the steps involved in the PPP process, as well as the significant complexity involved in shaping the PPPs.

In Ethiopia and Ghana, AHDP worked within the PPP legal frameworks, but because of lack of Ministry of Health (MOH) experience (Ethiopia) or new laws (Ghana), many respondents recognized that there was a steep learning curve for all concerned.

‘The whole PPP arrangement is now being done in accordance with the law. And the PPP law is very strict…Unlike before that there is no law and it is more about management discussions and management decides what they think is best for the institution and all those things. Now, you are going strictly in accordance with the provision of the law. Because it is the first of its kind, managing it and all those things is a little problematic. Because if there is precedence, it makes it easier.’ (Government, Ghana, 04)

Unfamiliarity with the laws also led to errors and missteps. The first request for qualification (RFQ) released by Ethiopia in December 2022 failed because it did not result in at least three bidders being successfully qualified (a requirement under the PPP framework). A further challenge was that the MOH was identified as the contracting authority, although under PPP guidelines, the implementing party (St Peter’s Hospital) should have been in this role. A second tender issued in March 2024, more than a year later, after consultations with potential bidders, included major changes to the scope of the contract specifically reducing the scope of the PPP from serving all public hospitals in Addis Ababa (*N* = 12) to only federal hospitals under the MOH (*N* = 6) to align with the federal PPP law as well as designating St. Peter’s Hospital as the contracting authority. The redesigned RFQ also included more accessible criteria, allowing a broader range of bidders to participate.

Complexity also added to transaction costs. In Ethiopia, to take the PPP forward several analyses and processes had to be managed concurrently, including updating public sector laboratory testing fees, exploring implications under the community (social) health insurance policy, establishing access to forex accounts for payment to the successful bidder, as well as legal and tax due diligence on the bidding companies.

#### Strategies and success factors

In Kenya, contracting arrangements were made under the Public Procurement and Asset Disposal (PPAD) Act of 2014—not the PPP Act. The decision to use the PPAD Act was driven largely by the fact that health is a county level responsibility in Kenya, and so PPPs for diagnostics were unlikely to lead to contracts of sufficient size to be processed under PPP laws. Although the PPAD act entailed multiple steps (including an expression of interest to obtain submissions, pre-qualification and determination of the minimum number of organizations that were required, RFP publication and sharing with pre-qualified organizations) it was not as burdensome as the PPP Act. While in theory the PPAD Act provides a quicker route to contract, the process from issuing requests for Expressions of Interest to signing a contract still took 16 months. Delays stemmed from negotiations regarding which form the partnership would take, and which policies governed how counties can implement revenue sharing and payments to private firms. Kenya’s 2022 general elections also slowed progress.

### Risk, vulnerabilities, and financing

#### Challenges

Project risk and financial issues proved to be major barriers to moving projects forward across all three contexts. Addressing these issues consumed considerable stakeholder time. Contrary to initial assumptions, governments were not interested in taking on debt (even at concessionary rates) to finance PPPs for diagnostic services. Ministries of Health (MOH) had little prior exposure to debt financing. When they first saw feasibility study findings, they were inclined to pursue large scale projects that would have required debt financing, but this was not supported by Ministries of Finance (Treasury). Reasons for lack of support from Ministries of Finance likely related to increased indebtedness in the region post-Covid; a reluctance to borrow for health service delivery projects compared to large infrastructure projects; and considerations around whether debt financing was necessary to cover capital expenditures of this scale. In practice, governments pursued PPP designs whereby private firms would invest capital of their own, supplemented by small-scale government investments.

Contrary to the initial project conceptualization, all three governments planned to finance the PPPs through a mix of user fee revenues and health insurance reimbursements, even though this would create an unpredictable revenue stream for the private provider.

I remember being quite surprised, actually, in Ghana, when the expectation…that this would be a program that would self-fund, or that would be funded primarily by the private sector, and that they [government] wouldn’t have to contribute. (Financing partner, Global, 2024.08.21)

Consequently, arrangements around the setting and management of user fees and insurance reimbursement mechanisms became critical. User fee schedules were bound by complex national regulations across all three countries. Often, these regulations had not been updated for a while and so were unlikely to garner adequate revenues for the private provider. Sometimes they did not even cover new tests on the expanded test menu:

One of the main reasons that the project took a lot of period is the price regulation, because as per Ethiopian regulations, under public service, if I'm not mistaken, around 150 tests were regulated, and the price is already stated, but this project is going to bring to around 400 tests, so around 300 tests has no price. (Health facility staff, Ethiopia 2024.08.29)

Another major point of risk negotiation was revenue share between the government and private partner. In Ghana, for example, the position of the Ministry of Finance was that given the upfront public investment in establishing infrastructure for the diagnostic facilities, they would expect to see revenues flow back to government. In Kenya, it was determined that 88% of revenues would go to the private contractor while 12% would go to the county due to the relatively heavy capital and recurrent spending allocated to the former party. However a few county officials felt that this was inappropriately low:

The percentage was, in my own opinion and in my own view, unfair. The partner is taking too much while we are giving too much. I felt that negotiation, though it was competitive… but I felt as if there should have been room for negotiation. (Government, Kenya, 2024.08.15b)

Other respondents in Kenya reported that the winning private partner was the only one of the three bidders who was willing to work within the existing user fee structure, and that it was likely that the profit margins were very small, justifying the proposed 88% allocation. In Ethiopia, the plan was for the private partner to retain all revenues, but the facility itself will revert to government ownership at the conclusion of the 13-year period of operation.

In Ghana and Kenya, reliance on user fees (paid to the public facility) to compensate the private provider led to practical concerns about how the funds would be ringfenced so that the private provider would get paid. This led to a further set of complex reforms, including the establishment of a Facility Investment Fund (FIF) in Tharaka Nithi, Kenya. The FIF law permits revenues collected to remain at the collecting facility, rather than going to the County Treasury. While the national FIF law was enacted later to allow such arrangements, at the time of putting the Tharaka Nithi contract in place, few counties had operationalized their own FIF laws. Even after the launch of the PPP, the FIF in Tharaka Nithi county was not fully functional, forcing the county to use its regular budget to pay the first invoice to the private partner.

In Ghana the National Health Insurance Authority (NHIA) specifies the use of case-based payment that does not separate diagnostics payments from the broader bundle of services. This made it difficult to guarantee revenue to the private provider. Substantial discussions took place on payment modalities with the most promising option being for the NHIA to pay individual facilities, which would then return funds to the national government to pay the diagnostics provider. This entailed considerable risk given the relative autonomy of healthcare facilities.

A broader concern related to the financial viability of insurance schemes across the countries, and the risks involved in relying on them for timely payment. For example, the NHIA in Ghana has had long-standing problems of late payment. While it has sought to address this challenge, late payment was seen by private providers as a material risk to PPP arrangements.

Obviously, we have to pay our vendors in time, maybe 30 days and 60 days. Now, if we don't get that payment in time, it means that we have to burn from our pockets. (Private partner, Global, 2024.08.28)

#### Strategies and success factors

The revised RFP issued by the Ethiopian government in 2024 incorporated alternative mechanisms to address risks facing potential private partners. Specifically, the RFP incorporated a risk mitigation mechanism to address delayed payments and unpredictability in revenue streams. In cases where revenue generation from public patients would fall short of expectations, the government would partially compensate the private provider. Conversely, if revenues were higher than anticipated, then the private provider would make additional payments to government. Such guarantees made the second RFP significantly more attractive.

To be frank, without those guarantees, we would not have proceeded, as a lack of volume would have been detrimental. (Private partner, Ethiopia, 2025.10.24)

The 2024 Ethiopia RFP also permitted charging higher user fees to ‘private patients’ to bolster the private partner’s revenues. In responding to the tender, the private partners had to specify the percentage increase over the standard public user fee schedule they were anticipating to charge private patients (defined to include patients who did not have community-based health insurance and were not referred through routine public facility channels). This RFP also stipulated periodic revision of user fees to account for inflation and rising cost of diagnostic services, however, private sector respondents noted that the process for price adjustment was unclear and ‘*poses a considerable challenge for the future’*. (Private partner, Ethiopia, 2024.10.18)

Magalhães et al identify modeling as an important strategy to address financial risks. For private partners, high test volumes were seen as important to drive down unit cost, optimize the use of existing equipment and permit automation of testing, all contributing to profitability. CHAI modeled predicted service volumes but much of the information required for robust modeling was unavailable For instance, service volumes are contingent on factors such as the number of patients currently seeking private services who might switch to the public sector as diagnostics became available; the reach of sample referral networks; the success of demand generation efforts; and broader policy changes affecting care seeking (e.g. social health insurance reforms), all of which were unclear and/or had weak empirical underpinnings. In Ethiopia, private partners were concerned about the unpredictable numbers of referrals from other hospitals connected to the network and how this might affect service volumes. As a result, the government developed referral agreements with other public hospitals and incorporated revenue guarantees.

### Market conditions

#### Challenges

In comparison to the Magalhães et al review, market conditions appeared to be a less important barrier to progressing projects. When RFPs were appropriately crafted, there was significant private sector interest. That said, the macro-economic context in Ethiopia made market conditions more challenging compared with the other two countries. Specifically, Ethiopia had limited foreign exchange reserves (meaning it would have been difficult for the government to pay a private contractor in foreign currency), strong controls on the repatriation of profits outside of the country, and a high risk of currency devaluation. Given the long duration of the contract and likely need to invest in new equipment during this time frame, private firms saw significant risks associated with devaluation.

#### Strategies and success factors

Some of the risks in Ethiopia were fortuitously addressed by a sweeping liberalization of Ethiopia’s foreign currency regime in June 2024. While this likely benefitted the AHDP PPP, it was clearly not a strategy promoted by the PPP.

In Ghana an interesting concern around market conditions came from multiple Ghana Health Service officials, who were worried that one large PPP would stifle competition. They instead proposed that the contract be broken into smaller packages for different regions to protect competition in the diagnostics market. As it was not thought viable to break up the contract, the decision was instead taken to reduce the number of regions participating.

You want to go through a process and give all your 16 regional facilities to a third party? It is highly risky, so why don’t we do one at the Northern belt and one at the Southern belt? As a pilot for like a year or two and see how the result will be before we roll out. It is health we are talking about here. It is not a supply of goods and whatever, this is healthcare delivery. (Government, Ghana, 04)

### Planning

#### Challenges

Table 4 shows the relative roles specified in the PPPs for government and private partners in Kenya and in Ethiopia (where contracts progressed) and also identifies the questions that the management teams from CHAI and government were addressing during the pre-project period regarding different aspects of project design. As can be seen, the PPPs touched on different elements of the health system, including staffing, referral systems, information systems, supply chain, and financing. With hindsight, planning across the different systems dimensions was likely inadequate. Several respondents from CHAI acknowledged that they had not initially recognized how complex the projects would be nor how central financing was to their ultimate success:

I think this is one of our failings here is that we underestimated the complexity of the financial aspect of this…This, from the start, was more of a financial solution than a healthcare solution. In many ways, it was a project that was housed inside our global diagnostics team, in theory, because we were running its diagnostics project. It's much more a health financing project than anything else. (Implementing Partner, Global 2024.08.06)

While the global project management team sought to conduct regular check in meetings and hold themselves to account by reporting progress against planned timelines, there were numerous factors outside of their control that made the timelines difficult to adhere to. Although Magalhães et al underscore the need for high level leadership, in practice many of the senior leaders among the partners supporting the project, both at the Gates Foundation and CHAI, moved on or became less actively involved over time.

**Table 4:** Relative roles of government and private partners under contractual arrangements in Ethiopia and Kenya.

Function/Role	Ethiopia	Kenya	Planning questions to be addressed
**Physical facility**			
Provision of site (building or room)	Public	Public	Which health care facilities should be included? Which entities own these facilities and can legally execute a contract on their behalf?
Development/renovation of laboratory	Private	Public	
**Staff**			
Provision of management staff	Private	Shared	Will existing lab employees be made redundant or be retained under a private contract? If government lab employees are retained then how will the private contract affect their conditions and terms of employment?
Provision of laboratory operations staff	Private	Public	
Training of laboratory staff	Private	Private	
**Equipment and supplies**			
Provision/purchase of new laboratory equipment	Shared	Private	Are existing contracts (e.g. equipment placement, or reagent supplies) in place and how will these be affected by the PPP?
Maintenance of laboratory equipment	Private	Private	
Provision of laboratory reagents and supplies	Private	Private	
**Service**			
Specimen referral from lower facilities	Private	Private	If specimen referral is included, then who provides transport and associated information systems?
**Laboratory information management system (LIMS)**			
Provision of information management system	Private	Private	To what extent are LIMS already in place? How will LIMS introduced by a private provider integrate with broader hospital information systems?
Ensuring interoperability with other systems	Private	Shared	
**Financing/payment for services**			
User fees collection	Private	Public	How will the scheme be financed? If the scheme is reliant on user payments then how will it (i) interface with social health insurance schemes and (ii) existing user fee schedules and regulations? Can providers be paid in forex?
Financial reporting	Private	Public	
Absorption of financing risk (e.g. non-payment)	Shared	Shared	
**Quality management**			
Ensuring attainment of accreditation	N/A	Private	Who will be responsible for ensuring quality of lab services?

LIMS, Laboratory Information Management System.

#### Strategies and success factors

Given the complexity of planning, there were gradual reductions in scope of the PPP in both Ethiopia and Ghana. For example, in Ethiopia, the initial vision was to cover Addis Ababa and scale nationwide thereafter. Instead the project shrunk from covering Addis Ababa to focusing on 12 public hospital laboratories, with the final version being structured to only equip one integrated diagnostic center that would serve only MOH hospitals in the city. Similarly, the Ghana project shrunk from plans to cover the entire country to covering half. Matching the scope and scale of the PPP to the needs and capacity on the ground revealed an important success factor.

### Stakeholder management

#### Challenges

Across all three countries, the PPPs stimulated political sensitivities due to the newness of the modality, limited government experience with the private sector, and a history of mistrust. In Kenya, political concerns were raised by members of the county assembly who thought the PPP was expensive and worried about optics of privatizing services vis-à-vis enhancing government capacity to deliver services. A variety of stakeholders in all three countries acknowledged the critical importance of garnering support from a wide variety of actors, especially in light of rapid turnover among senior officials, and how time consuming the process was.

You really needed to carry every player with you, every step of the way because if we just started without disseminating the needs for this to the people, and even collecting the data on the needs assessment, then somebody might have accused us of bringing in a private practitioner within the county, and …you know… politics. (Health facility, Kenya, 2024.08.15)

Multiple reasons underlay resistance to the PPP. In Ghana, there were initial concerns about the PPP’s impact on public sector lab technician jobs. In Ghana and Kenya, a small number of respondents reflected on how the PPP would close leakages in the system, including reducing opportunities for government staff to refer patients to private laboratories they owned.

The project had too many vested interests. One, the labs in the regions. A lot of those labs [centers] have many private labs that were there. All of a sudden you were taking my revenue. There were a lot of issues with it. (Implementing Partner, Ghana, 2024.08.09)

While governments were enthusiastic about the PPP, there was tension in their relationship with private partners (described below under organizational culture).

Another key observation was the limited capacity to manage complex processes involved in pre-project negotiations and on-project implementation. Limited capacity across health and finance ministries caused delays. While CHAI provided technical support to the project it acknowledged that there were domains, particularly financing, where it under-estimated the complexity and need for support, as well as specific capacities (primarily related to legal issues and PPPs) where it found it difficult (and expensive) to secure relevant expertise.

It was very hard to secure a global legal expert on PPP. It was a very expensive project. I remember someone requested almost the entire annual budget of the AHDP country office for that kind of work…If you want to realize a PPP, you need more technical resources. (Implementing partner, Ethiopia 2024.08.30)

#### Strategies and success factors

Across all three countries, CHAI played a mediator type role, though not formally defined as such. This was especially important during the negotiation process between the Tharaka Nithi County Government and the private contractor in Kenya. The role also helped progress discussions whenever differences occurred during the project implementation stage.

‘I'm not saying it has to be CHAI but a neutral third party that can be able to balance the goals and the motivations of both of them and bring an even playing field […] There's a lot of managing that I thought after we signed the contract would end. It only increases when implementation starts.’ (Implementing Partner, Kenya)

Beyond supporting PPP process for initiating and implementing a PPP, a neutral actor can help diffuse tensions between government and private actors.

A third party mediator may be helpful in addressing capacity gaps. The Ethiopian Government emphasized the importance of CHAI supporting to the PPP, particularly in building the MOH’s capacity to plan the PPP and develop tender documents. The Ministry of Finance, however, supplemented the support it received from CHAI with technical assistance from Expertise France, a long-term technical assistance provider with substantial PPP experience (who supported the drafting of the second RFP). This suggests another important management strategy, focused on leveraging highly specialist expertise.

### Organizational culture

#### Challenges

Perhaps because project implementation had only proceeded in one county in Kenya, we found limited evidence of differences in organizational culture between public and private sectors impacting the PPPs, however, there were two domains where lack of alignment was apparent.

The first relates to differing approaches to timelines. While there were frequent delays on the government side in terms of processing or reviewing documents, this would not lead to extension of the deadline for the private partner, but rather the private partner’s work was compressed to fit the overall timeline. Understandably this created frustration on the part of private partners.

‘Again, as I said that, again, I think the clarity, see what happens, that when it comes to private and the public, there's always a disconnect in terms of decision making, in terms of following the right turnaround time for everything, whether it's your payment, whether to respond to your email or either to, timelines is a challenge.’ (Private Partner, Kenya)

The second challenge concerned equity. Although AHDP was not specifically designed to benefit the poorest, it intended to promote equity through increasing access to, and quality of a wider range of diagnostics for the general population, thus decreasing out-of-pocket payments for diagnostics. While this sentiment was echoed by many respondents, others voiced concern over how the PPP should be managed to protect equity.

Our big goal was to reduce catastrophic expenditure. The number of people pushed to poverty or below poverty because of huge medical expenses will reduce, at least in Addis Ababa. Because a single MRI image now at the private sector is around 8000 Ethiopian Birr (US$58). While at this center, it would be around 1200 Birr. It’s almost 70, 80% reductions. (Implementing partner, Ethiopia, 2024.08.30)

The quality of service will improve, but there is also a challenge working with the private sector. The interest of private, even if they are efficient, even if they have their own technology, expertise and so on, they have their own motive. They are profit-making organizations, so in that regard, equity may not be realized. The equity issue will be addressed by government. (Government, Ethiopia, 2024.08.28)

#### Strategies and success factors

Differences in organizational culture between government and private sector companies were not fully resolved within AHDP. While governments sought to protect access to care by capping user fees to be paid to private contractors, they were not prepared to provide additional funding to support the PPPs. As a consequence, and given limited implementation at the time of this study, the extent to which AHDP has or will lower financial barriers to diagnostics remains unclear.

## Discussion

Our research found that management of PPPs under the AHDP project aligned with challenges identified in the broader PPP literature outside of health (see summary in Table 5). Certainly, intensive negotiations and high transaction costs; difficulties managing risks and financing; the need for complex planning; and challenging stakeholder management all played a role in slowing progress on AHDP. On the other hand, market conditions were less of an issue compared to the broader literature, perhaps reflecting the lower level of capital investment required for this (and many other health sector projects), especially when compared to infrastructure projects. While we observed differences in organizational culture, at the time the study closed out, these had not created substantive obstacles to progress, though implications for equity under new organizational arrangements remain an important and unresolved consideration. A further factor, less visible in the broader PPP literature but prominent for AHDP, was low capacity across government and technical assistance partners to support health PPPs. Limited government capacity for health PPPs has been observed elsewhere (Ovcharova and Grabowska 2022, Dove et al. 2025). Our findings reinforce this. Finally, while Magalhães et al distinguish between project phases (pre-project, on-project), we found such distinctions difficult to maintain in practice, with pre-project challenges persisting into the on-project phase.

**Table 5:** Summary of project management challenges, strategies and success factors observed in the African health diagnostics platform project*.

	Construct & definition	Challenges	Strategies	Success factors
**Pre-Project**	**Transaction costs**—time & effort spent on finding contractors, researching options, getting quotes, negotiating terms and finalizing the contract	Limited understanding of complex PPP Acts Intensive and time-consuming research & negotiations to arrive at contracts Need to manage PPP laws concurrently with other policies and acts (e.g. updating user fee schedules)	Contracting under simpler public procurement acts (Kenya)	Smaller contracts in Kenya due to decentralization and county-led health services.
	**Risk, Vulnerabilities & Financing (Pre-project & on-project)**—potential events that could affect project viability, underlying weaknesses that increase exposure to these risks and funding for the project	Governments unwilling to take on debt to finance health PPPs Projects depended on user fee and health insurance financing pushing risk to private sector Outdated user fee schedules (Ethiopia) Concerns regarding payment risk (late or no payment)	Innovative risk mitigation mechanisms to compensate private provider for low use, and protect against payment risk (Ethiopia) Agreements to drive up referrals from other facilities so as to increase service volumes (Ethiopia) Modeling of risks and financing—weak given lack of research base	Low levels of indebtedness (high indebtedness in study countries undermined PPPs)
	**Market conditions**—the economic, financial & industry environment and how it affects the private sector’s appetite for PPPs	Challenges related to forex in Ethiopia	Steps to disaggregate large contracts to preserve competition in the market (Ghana)	Relatively favorable investment environment in Ghana and Kenya. Improved environment in Ethiopia with 2024 financial liberalization Substantial industry interest in diagnostic PPPs
**On-Project**	**Risk, Vulnerabilities & Financing**—potential events that could affect project viability, underlying weaknesses that increase exposure to these risks and funding for the project	Flaws in project models and estimations Unexpected political and economic vulnerabilities Ensuring adequate funding to sustain project	External reviews of planning and project models Regulatory frameworks to minimize risks from political and economic instability	
	**Planning**—coordination of the technical, financial and institutional aspects of the project to ensure it is delivered and operated according to the PPP terms	Project planning did not adequately take account of project complexity	Reductions in scope of project to match capacity on the ground (Failure to) maintain project management team with high level leadership	
	**Stakeholder Management**—ongoing process of identifying, engaging and coordinating stakeholders to manage expectations, address concerns and support effective PPP delivery	Need for ongoing high levels of stakeholder engagement throughout the life of the project to sustain project support History of mistrust between public and private actors Concerns that PPPs might undermine personal interests (minority view)	Use of neutral third party mediator Engagement of specialist capacity to support Failure to use structured stakeholder management approaches	
	**Organizational culture**—shared values, norms, behaviors and ways of working, and the degree of alignment between public and private sectors	Private sector sought hard deadlines whereas timelines were more ‘elastic’ in the public sector. Public sector actors valued equity, whereas less central for private firms.		Level of trust between parties

^*^Points apply to all three countries unless noted otherwise.

PPP, public–private partnership.

Partners in AHDP identified and pursued some promising strategies for supporting better PPP management. These included:

- Using innovative mechanisms to better distribute risk between government and the private sector (e.g. during the second RFP in Ethiopia);
- Engaging a neutral trusted third party to assist with managing relations between government and private sector actors;
- Engaging highly specialized PPP expertise to support design processes (Ethiopia);
- Breaking down larger PPP projects into smaller ones to reduce complexity and prevent monopoly;
- Operating under public sector procurement rules rather than PPP laws (where project size permitted) (Kenya).

While some of these practices appear promising and have precedence (see e.g. Torchia et al. 2015, Vian et al. 2015, Wafula et al. 2022), they may present different risks for sub-Saharan African health systems. For example, public procurement laws rarely offer the same protection from political interference that PPP laws provide. Similarly, while breaking PPPs into smaller projects may make them easier to implement, doing so presents additional risks linked to low probability of scaling (Kula and Fryatt 2014).

Beyond the strategies that were employed in AHDP, our study insights suggest additional practices that could help strengthen the management of health sector PPPs namely:

### Transaction costs

PPP projects are, by definition, negotiation heavy, but this was exacerbated under the AHDP project by public sector actors’ lack of familiarity with PPP laws and processes. This led to missteps and decision reversals. Technical assistance providers helped, but were unable to fill all gaps, or were at times, late filling them. Building understanding of PPPs and how they work in the health sector is a priority for addressing many of the challenges identified in this study. There are a number of online guides to PPPs in the health sector and best practice documents (Asian Development Bank 2013, World Economic Forum 2021) but there is a dearth of regular training courses, communities of practice or online repositories of resources that can help health sector stakeholders navigate PPPs more effectively. Initiatives like the Open Phences Hub (‘Openphences - Bridging healthcare in public and private sectors’ n.d.) provide some support to countries in the region, but more effort is urgently needed to develop stronger institutional mechanisms and training materials to build capacity at scale.

### Risk, vulnerabilities and financing

Training and developing reference materials that set out alternative approaches to risk management in PPPs would help address management challenges similar to those found in AHDP. Another strategy entails developing robust models that demonstrate risk distribution and how this shifts with alternative contractual arrangements. While such models would be instructive, they may be difficult to develop given the lack of evidence about many of the factors needed to model diagnostic PPPs. For example, data from the region about current patterns of diagnostic utilization; evidence on patients’ willingness and ability to pay for diagnostics; the price sensitivity of demand for diagnostics; how best to structure diagnostic referral networks; and diagnostic financing are all lacking. Studies of these topics could improve modeling and forecasting and inform decision-making on how best to engage the private sector in strengthening diagnostics.

### Planning

Developing and implementing a PPP for a discrete component of the health system might initially appear relatively straightforward, however as the AHDP project revealed, even PPPs for a single health system component (diagnostics) can turn out to have major implications for the health system as a whole, including patient journeys, health financing, and facility management. Partners need to anticipate, and plan for complexity that comes with PPP projects. Traditional, linear planning approaches may fail to accommodate contingencies within PPPs, and are unlikely to adequately reflect linkages with other sub-systems. Systems-thinking based planning approaches like causal loops or process mapping can help to identify relevant sub-systems, risks, assumptions and contingencies (Peters 2014, Khalil and Lakhani 2022). Planning approaches need to be dynamic and adaptive, incorporating learning as the project proceeds, and assumptions are proven (or not).

### Stakeholder management

Public sector uptake and scaling of private sector solutions requires investment in building cohesion across ecosystem actors (Caldwell et al. 2017, Wafula et al. 2022). In AHDP, demonstrating the social value of the project to different actors was a challenge across all three countries, yet an important driver of PPP success (Caldwell et al. 2017). Political sensitivities and historical mistrust between actors meant that it was necessary to engage a wide variety of stakeholders on a continuing basis in order to secure and sustain buy-in to the project.

Although AHDP implementing teams thought systematically about which stakeholders to engage, they neither developed formal stakeholder management plans nor conducted political economy analyses. A mapping of political interests and sensitivities, as a means to identify potential opponents and address concerns early would have been beneficial. The development of a regularly updated stakeholder management strategy should ideally be baked into PPP management processes. While some papers address the political economy of PPPs in the global north (Flinders 2005, Whiteside 2011) or global level PPPs (Reich 2002, Ruckert and Labonté 2014), more in-country research is needed to understand the likely position of different stakeholders on PPP proposals, and how to shape PPP designs to accommodate competing interests.

### Limitations

Despite the extended engagement of the research team over a five-year period, we were unable to trace the PPP projects through to their final form. The number and pattern of respondents in the final round of interviews differed across countries and appears uneven, however, based on the first round of interviews, the research teams had a solid of understanding as to who could provide valuable information and all of the interviews conducted were rich in content. Also, while interviewers attempted to probe respondents to fully understand their perspectives, we acknowledge that response bias might have influenced our data, due both to social desirability and also, particularly on the part of private sector respondents, a desire to shape market conditions, or protect a firm’s interests.

## Conclusions

There continues to be much discussion about the promise of PPPs in the health sector of LMICs, but as reported elsewhere, the evidence base underpinning this is weak. Our paper demonstrated the significant challenges involved in managing health PPPs at scale. While PPPs may indeed hold substantial promise, stakeholders wishing to invest in them need to prepare for intensive negotiations, complex planning, strong technical support, and sophisticated political management. Although PPPs will need to be tailored to country contexts, facilitating peer-learning networks among countries would support leapfrogging of challenges and the spread of good practices. Even if the evidence base supporting PPPs in health is fragile, given ongoing shifts within the development field towards more commercially-oriented practices and investments (Unger et al. 2025), empowering key stakeholders including both government officials and technical assistance providers, with a stronger understanding of how PPPs work, as well as practical tools to help them navigate PPP negotiations will be critical.

## Data Availability

The data underlying this article will be shared on reasonable request to the corresponding author who will coordinate as appropriate with lead country researchers.

## Reflexivity statement

This paper was authored by a diverse team including representatives of four different institutions namely (1) Johns Hopkins University that held the primary grant ad led global level interviews and analysis (2) the International Institute of Primary Health Care in Ethiopia (3) the University of Ghana and (4) Strathmore University in Kenya. At the beginning of the work of the consortium a set of principles for working collaboratively were developed collectively by the partners that have guided both the research and the development of outputs. The team involved in this work brought diverse academic backgrounds (clinical medicine, public health, health economics and policy, and business) as well as varied initial positions on the advantages and disadvantages of private sector engagement in health care. As described in more detail in the methods section and the table of author contributions, country teams spearheaded data collection and analysis in each of their own countries and JHU at the global level, while seeking to ensure similar overarching frameworks to facilitate comparative analysis. Findings reported here were arrived at through multiple discussions involving all team members. Additional papers, both country-specific and comparative, taking more focused lenses to the data are planned and will be led by different authors from the team.
